# Role of IMRT/VMAT-Based Dose and Volume Parameters in Predicting 5-Year Local Control and Survival in Nasopharyngeal Cancer Patients

**DOI:** 10.3389/fonc.2020.518110

**Published:** 2020-09-24

**Authors:** Nicola Alessandro Iacovelli, Alessandro Cicchetti, Anna Cavallo, Salvatore Alfieri, Laura Locati, Eliana Ivaldi, Rossana Ingargiola, Domenico A. Romanello, Paolo Bossi, Stefano Cavalieri, Chiara Tenconi, Silvia Meroni, Giuseppina Calareso, Marco Guzzo, Cesare Piazza, Lisa Licitra, Emanuele Pignoli, Fallai Carlo, Ester Orlandi

**Affiliations:** ^1^Radiotherapy Unit 2, Fondazione IRCCS Istituto Nazionale dei Tumori di Milano, Milan, Italy; ^2^Prostate Cancer Program, Fondazione IRCCS Istituto Nazionale dei Tumori di Milano, Milan, Italy; ^3^Medical Physics Unit, Fondazione IRCCS Istituto Nazionale dei Tumori di Milano, Milan, Italy; ^4^Head and Neck Medical Oncology Unit, Fondazione IRCCS Istituto Nazionale dei Tumori di Milano, Milan, Italy; ^5^Department of Radiology, Fondazione IRCCS Istituto Nazionale dei Tumori di Milano, Milan, Italy; ^6^Department of Otolaryngology, Head and Neck Surgery, Fondazione IRCCS, Istituto Nazionale dei Tumori di Milano, Milan, Italy; ^7^Department of Oncology and Hemato-Oncology, University of Milan, Milan, Italy; ^8^Radiotherapy Unit 1, Fondazione IRCCS Istituto Nazionale dei Tumori, Milan, Italy

**Keywords:** nasopharyngeal carcinoma (NPC), intensity-modulated radiation therapy (IMRT), gross tumor volume (GTV), dose-volume parameters, outcomes, nomogram, volumetric-modulated arc therapy (VMAT)

## Abstract

**Objective:** This study aimed to look into the relationship between intensity-modulated-radiotherapy (IMRT)- or volumetric-modulated-arc-therapy (VMAT)-based dose–volume parameters and 5-year outcome for a consecutive series of non-metastatic nasopharyngeal cancer (NPC) patients (pts) treated in a single institution in a non-endemic area in order to identify potential prognostic factors.

**Materials and methods:** A retrospective analysis of consecutive non-metastatic NPC pts treated curatively with IMRT or VMAT and chemotherapy (CHT) between 2004 and 2014 was conducted. One patient was in stage I (0.7%), and 24 pts (17.5%) were in stage II, 38 pts (27.7%) in stage III, 29 pts (21.2%) in stage IVA, and 45 pts (32.8%) in stage IVB. Five pts (3.6%) received radiotherapy (RT) alone. Of the remaining 132 pts (96.4%), 30 pts (21.9%) received CHT concomitant to RT, and 102 pts (74.4%) were treated with induction CHT followed by RT-CHT. IMRT was given with standard fractionation at a total dose of 70 Gy. Clinical outcomes investigated in the study were local control (LC), disease-free survival (DFS), and overall survival (OS). Kaplan–Meier (KM) analysis was performed for the outcomes considering dose and coverage parameters, staging, and RT technique.

**Results:** Overall, 137 pts were eligible for this retrospective analysis. With a median follow-up of 70 months (range 12–143), actuarial rates at 5 years were LC 90.4, DFS 77.2, and OS 82.8%. For this preliminary study, T stage was dichotomized as T1, T2, T3 vs. T4. At 5 years, the group T1–T2–T3 reported an LC of 93%, a DFS of 79%, and an OS of 88%, whereas T4 pts reported LC, DFS, and OS, respectively, of 56, 50, and 78%. Pts with V95% > 95.5% had better LC (*p* = 0.006). Pts with D99% > 63.8 Gy had better LC (*p* = 0.034) and OS (*p* = 0.005). The threshold value of 43.2 cm^3^ of GTVT was prognostic for LC (*p* = 0.016). To predict the risk of local recurrence at 5 years, we constructed a nomogram which combined GTVT with D99% relative to HRPTV.

**Conclusions:** We demonstrated the prognostic value of some dose–volume parameters, although in a retrospective series, this is potentially useful to improve planning procedure. In addition, for the first time in a non-endemic area, a threshold value of GTVT, prognostic for LC, has been confirmed.

## Introduction

Intensity modulated radiation therapy (IMRT) is an important milestone in the management of nasopharyngeal carcinoma (NPC), providing lowered frequency of serious radiation-induced late toxicities without compromising local control (LC) and survival compared to previous radiotherapy (RT) techniques (1). A radiation–dose response for NPC has been demonstrated with dose escalation of IMRT-based therapy by using both additional sequential boost over 66 Gy (2) and increasing biologically equivalent doses up to over 70 Gy by simultaneous integrated boost (SIB) IMRT (3–5). Still, the outcomes after IMRT remain unsatisfactory in T4 tumors, most of all because their proximity to critical neurological structures compromises planning target coverage and therefore undermines LC. In the most recently published NPC series, concerning endemic regions and providing the longest follow-ups to date, T1–T3 diseases had excellent LC unlike T4 lesions (6–8). Also, T4 patients are well-known to be at high risk of developing distant metastases or even dying, so they require an aggressive systemic approach by adding induction and/or adjuvant chemotherapy (CHT) (9–12). Assuming that CHT may make up for coverage defects of the target volume, it is still hard to set a benchmark for dosimetric adequacy. As a matter of fact, data correlating IMRT-based dose parameters and outcome are scanty, making it difficult to identify their potential prognostic role.

Even in the IMRT era, the therapeutic choice in NPC cases is primarily driven by the tumor–node–metastasis (TNM) staging system. Several previous studies from endemic regions have described some indicators resulting from imaging, i.e., gross tumor volume (GTV) of primary tumor as defined by magnetic resonance imaging (MRI), as prognosticators with a potential to increase the precision of TNM criteria (13–15). However, in low-incidence areas, the impact of primary tumor GTV has never been investigated.

The first aim of this study was to look into the relationship between IMRT- or volumetric-modulated-arc-therapy (VMAT)-based dose and volume parameters and outcome at 5 years for a consecutive series of NPC patients treated in a single institution in a non-endemic area, in order to identify potential prognostic factors. Secondly, we aimed to establish novel target volume constraints for planning optimization.

## Materials and Methods

### Study Population

From May 2004 to April 2014, 160 consecutive patients with non-metastatic NPC received curative IMRT or VMAT with or without CHT at our institution. Eligibility criteria for this retrospective analysis were as follows: a minimum follow-up of 5 years, MRI before any treatment, and availability of full clinical and dosimetric data. Thus, 137 patients out of 160 met the inclusion criteria and were considered for this analysis. Over the course of the analysis, all patients were restaged according to the AJCC 2010 staging classification seventh edition (16). This study was approved by the ethics committee of the “Fondazione IRCCS Istituto Nazionale dei Tumori di Milano,” and all patients signed an informed consent to use their data for research purposes in line with the policy of our institution. The study was performed in accordance with the ethical standards laid down in the 1975 Declaration of Helsinki and all subsequent revisions.

### Treatment

#### Radiotherapy

Details of target volume delineation and IMRT planning and delivery procedures have been previously reported (17). There were no changes in delineation strategies during the entire study period. In synthesis, GTV included nasopharyngeal primary tumor and involved lymph nodes as demonstrated by clinical, endoscopic, and imaging data (MRI and ^18^F-FDG PET/CT), i.e., global GTV (gGTV). For this study purpose, we retrospectively contoured the following GTVs separately: nasopharyngeal primary tumor (GTVT) and involved nodes (GTVN), deriving from the sum of involved lymph nodes including retropharyngeal involved nodes (GTVNRP) and involved nodes other than retropharyngeal (GTVNNRP). For patients receiving induction CHT (iCHT), all GTVs were contoured on pre-CHT magnetic resonance images.

A high-risk (HR) clinical target volume (CTV), including both GTVT and GTVN with an anisotropic margin ranging from 0 to 25 mm taking into account subclinical disease, and a low-risk (LR) CTV have been defined for all patients, whereas an intermediate-risk (IR) CTV was contoured in selected cases. Planning target volumes (PTVs) were generated by adding a 3-mm margin to corresponding CTVs, i.e., high-dose (HD) PTV (HDPTV), intermediate-dose (ID) PTV (IDPTV), and low-dose (LD) PTV (LDPTV). The goal for HDPTV was usually to deliver at least 95% of the prescribed dose (PD) to at least 95% of HDPTV without exceeding tolerance doses to neurological organs at risk (n-OARs).

During the study period, RT was given with conventional fractionation (2–2.12 Gy per fraction) according to some technical approaches. From 2004 to 2009, IMRT was routinely delivered with the step-and-shoot technique with a 7-coplanar 6-MV photon beam arrangement. Two approaches were used: (i) a purely sequential (SEQ) approach, with conventional fractionation (2 Gy per fraction) up to 50–54 Gy to LDPTV and 70 Gy to HDPTV; and (ii) a mixed approach SEQ-SIB, which comprised a first phase of 30 fractions of 1.8 Gy to LDPTV (PD = 54 Gy) and 2–2.12 Gy to HDPTV (PD = 60–63.6 Gy) followed by a boost of five 2-Gy or three 2.12-Gy fractions to HDPTV (PD = 70 Gy). When defined, IDPTV received 60–66 Gy with 2-Gy fractions in either SEQ or SEQ-SIB mode. Since 2009, VMAT, with two to four coplanar arcs, has been gradually implemented in our practice, eventually becoming the standard technique for this disease in our center. A SIB regimen was given in 33 fractions with a PD of 69.96 and 56.1 Gy to HDPTV and LDPTV, respectively. When defined, IDPTV was planned to receive 59.4 Gy in 33 fractions.

For PTV coverage, the following parameters were recorded unrelated to the RT technique: minimum dose (D99%), maximum dose (D1%), mean dose (DMean), and the percentage of target volume receiving 95% (V95%) and 100% (V100%) of its PD.

#### Chemotherapy

Patients at stages I and IIA received exclusive RT, whereas patients at stages IIB–III–IV received concomitant platinum-based CHT. iCHT with docetaxel, cisplatin, and 5-fluorouracil was added to patients with a potential higher risk of distant metastasis, according to our previously reported institutional policies (18).

### Follow-up

After RT completion, patients were clinically evaluated at predefined intervals, typically every 3–6 months for the first 3 years and annually thereafter. MRI and ^18^F-FDG-PET were prescribed on a regular basis and when deemed necessary according to patients' disease status.

### Statistical Analysis

Survival and recurrence time observations were plotted according to the Kaplan–Meier method starting from the first day of treatment (CHT or RT, whichever came first). Overall survival (OS) was defined as the time from treatment start until death from any cause. Disease-free survival (DFS) was defined as the time from treatment start to disease recurrence or death. LC was defined as the time from treatment start until local recurrence. Actuarial 5-year rates of LC, DFS and OS were calculated.

The following dose and volume parameters for HDPTV were studied as potential prognostic factors of OS, DFS, and LC: D99, D1%, DMean, V95, and V100% of the PD. This is because recurrences occurred mostly “in field” or were classified as “marginal” to HDPTV (17). Moreover, GTVT, GTVN, GTVNNRP, GTVNRP, and RT technique—conventional IMRT vs. VMAT—were investigated.

We looked into correlations between HDPTV parameters and LC and not LRC because difficulties in target volume coverage are linked in particular with primary tumor extension (in particular T4) rather than nodal disease.

The maximal chi-square method was used to determine the optimal cutoff values for the association between continuous parameters and clinical outcomes. Thus, each feature has been dichotomized according to the quartile of their distribution closest to the derived best cutoff. Kaplan–Meier actuarial curves were generated for the significant parameters (with a *p* < 0.05 resulting from the *t*-test), and a log-rank test was used to verify if curve separation was statistically significant (*p* < 0.05) even considering the time dependence of the corresponding survival curve. Univariable Cox regression analysis was performed to estimate the hazard ratio (HR) associated with the variables.

Univariate and multivariate Cox regression analyses were finally performed for 5-year rates of OS, DFS, and LC including all the dosimetric variables and the following clinical factors: T stage, N stage, and overall stage. The latter were dichotomized as T1–T2–T3 vs. T4, N0–N1–N2 vs. N3a–N3b, and stages I–II–III vs. IVA–IVB. For that analysis, patients with an event within 5 years were selected together with those who are event free and had a minimum follow-up of 5 years. Finally, a nomogram was computed starting from the Cox proportional hazard regression model.

All statistical analyses were performed in the KNIME environment (KNIME GmbH, Germany) coupled to R software (www.r-project.org).

## Results

Clinical and treatment-related characteristics of the 137 patients included in this study are shown in Table 1. One patient was in stage I (0.7%), and 24 patients (17.5%) were in stage II, 38 patients (27.7%) in stage III, 29 patients (21.2%) in stage IVA, and 45 patients (32.8%) in stage IVB. In particular, 39 patients (28.5%) were stage T4.

**Table 1:** Clinical and treatment-related characteristics of the 137 patients included in the study.

		**Total**	**Percentage (%) or range**
All patients		137	100
Sex	Male	96	70.1
	Female	41	29.9
Age (median)		49	18–92
ECOG	0–1	131	95.6
	2	6	4.4
Histology	Keratinizing squamous cell carcinoma (WHO type 1)	1	0.7
	Non-keratinizing (WHO type 2)	2	1.5
	Undifferentiated (WHO type 2)	134	97
Neck surgery^*^		22	16.1
Stage T	1	48	35.0
	2	31	22.6
	3	19	13.9
	4	39	28.5
Stage N	0	4	2.9
	1	29	21.2
	2	59	43.1
	3a	14	10.2
	3b	31	22.6
Stage	I	1	0.7
	II	24	17.5
	III	38	27.7
	IVA	29	21.2
	IVB	45	32.9
Treatment	RT alone	5	3.6
	RT-CHT	30	21.9
	iCHT + RT-CHT	102	74.5
RT technique	IMRT	73	53.3
	VMAT	64	46.7
b-EBV-DNA^**^	Median	510	0–150,075
	Negative (pts)	36	29.3
	UQ + Q + Q^+^ (pts)	87	70.7

*ECOG, Eastern Cooperative Oncology Group*.

* *Excisional biopsy or functional or radical laterocervical dissection*.

** *Only 123 patients out of 137; b-EBV-DNA was stratified into four groups: negative (b-EBV-DNA = 0); UQ, positive but unquantifiable (0 < b-EBV-DNA <102 copies per milliliter); Q, positive and quantifiable (102 ≤ b-EBV-DNA ≤ 15 × 102 copies per milliliter); Q^+^, strongly positive and quantifiable (b-EBV-DNA > 15 × 102 copies per milliliter). For details, see Alfieri et al. (18)*.

As for CHT, five patients out of 137 did not receive CHT according to disease stage. Thirty patients out of 137 received concomitant platinum-based CHT with a median cumulative platinum dose of 225 mg/sm (range 150–300 mg/sm; mean 244 mg/sm). iCHT was administered in 102 out of 137 patients followed by concomitant CHT; 100 patients received TP schedule with or without 5FU, and two patients received PF schedule. All patients continued with platinum-based CHT concomitant to RT, with a median cumulative platinum dose of 250 mg/sm (range 50–300 mg/sm; mean 235 mg/sm).

**Figure 1:**
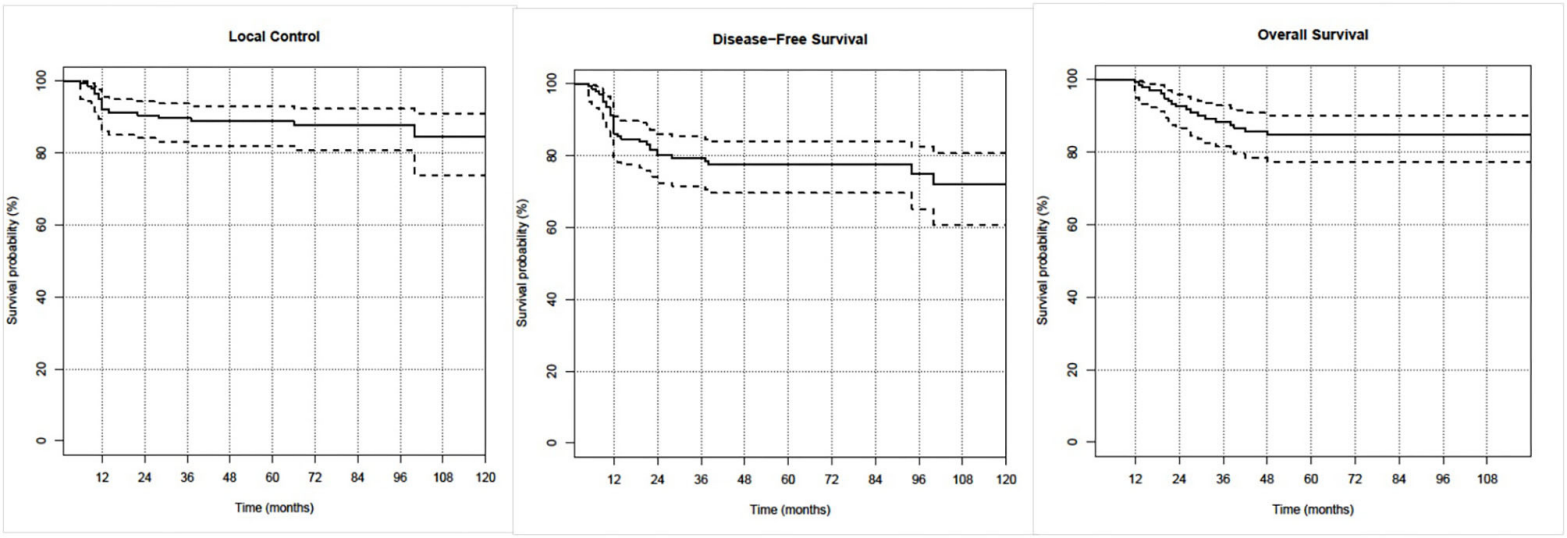
Kaplan–Meier curves of the three investigated clinical outcomes.

The median follow-up period was 75.2 months (range: 12–141.1 months). Actuarial rates at 5 and 8 years were, respectively, 90.4 and 88.1% for LC, 77.2 and 74.3% for DFS, and 82.8 and 82.8% for OS. OS, DFS, and LC curves for the entire population are shown in Figure 1.

Sixteen out of 137 patients developed distant metastases (11.7 %) during the follow-up period, with three of them showing also a local recurrence of the disease.

Dose and volume statistics for the entire population are shown in Table 2. *t*-test results for parameter selection are reported in the (Supplementary Table 1) together with the distribution of the dosimetric variables selected for survival analysis in terms of mean value and standard deviation, stratified according to OS, DFS, and LC (Supplementary Table 2).

**Table 2:** Dose and volume statistics.

**Variable**	**Mean ± SD**	**Range (min–max)**	**I quartile**	**Median**	**III quartile**
GTVT (cm^3^)	33.3 ± 34.8	2.2–173.3	7.6	21.1	43.2
GTVN (cm^3^)	39.9 ± 47.9	0.2–332.7	9.0	25.1	55.2
GTVNRP (cm^3^)	5.1 ± 8.1	0.2–43.3	2.2	4.0	9.7
GTVNNRP (cm^3^)	34.6 ± 45.7	0.5–326.1	7.5	21.0	47.7
V95% (HDPTV) (%)	93.5 ± 15.0	16.1–100.0	95.5	98.4	99.7
V100% (HDPTV) (%)	60.3 ± 24.7	0–99.5	47.8	62.5	79.6
D1% (HDPTV) (Gy)	74.3 ± 2.5	67.8–84.3	72.9	74.3	75.4
D99% (HDPTV) (Gy)	65.2 ± 3.0	53.6–70.4	63.8	65.9	67.5
DMean (HDPTV) (Gy)	70.3 ± 1.7	65.1–75.7	69.8	70.4	71.1
HDPTV Volume (cm^3^)	295.1 ± 140.2	58.1–833.0	188.0	289.0	378.0

The results of univariate Cox regression analysis for LC, DFS, and OS on the whole set of parameters are reported in Supplementary Tables 3–5.

As for RT parameters, we found that V95, V100, D99, DMean relative to HDPTV and GTVT, and RT technique were significant for LC, and V95% relative to HDPTV and RT technique were significant for DFS. Finally, V95, V100, D99% relative to HDPTV, and RT technique were significant for OS.

**Figure 2:**
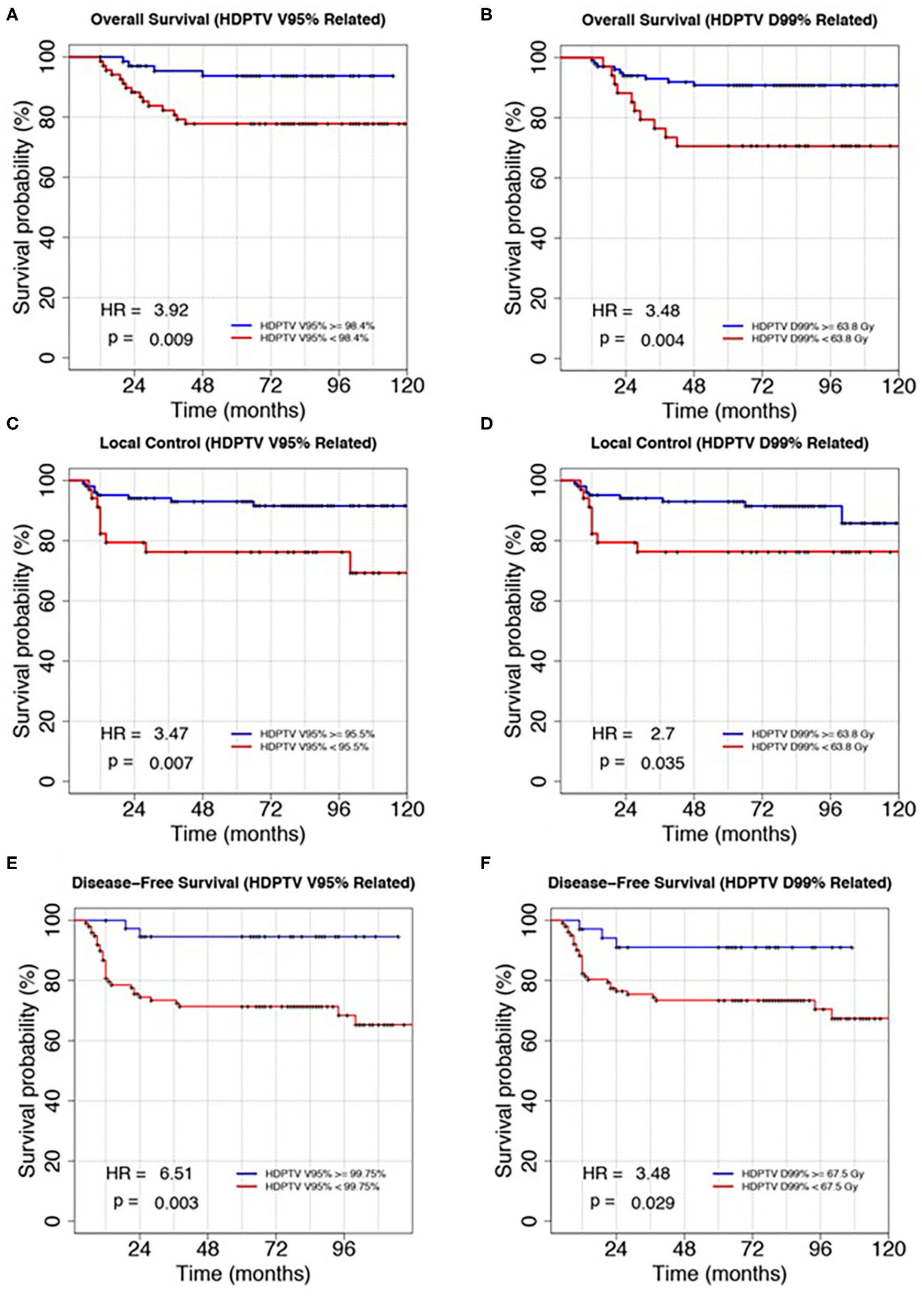
Kaplan–Meier plots of overall survival, local control, and disease-free survival discriminating patients according to the HDPTV V95% **(A,C,E)** and HDPTC D99% **(B,D,F)**. Variables were dichotomized according to the method described in the main text. Hazard ratio (HR) and log-rank *p*-value were reported in the bottom left corner of each plot.

Figure 2 shows the survival curves for the independent prognostic parameters common to all the analyzed outcomes: HDPTV V95, V100, and D99%. Due to the natural correlation between V95 and V100%, we decided to select HDPTV V95% for its major clinical–dosimetric value, which also had the lowest *p*-value. Variables were then dichotomized as described before (quartile closest to the best cutoff). In particular, HDPTV V95 and D99% were dichotomized according to the second and first quartiles of their distribution, respectively, for OS, the third quartile for DFS, and the first quartile for LC. HR and log-rank *p*-value for the new dichotomized variables were also reported in the figure.

In particular, different cutoffs of dose–volume parameters were found to be prognostic for the three outcomes considered: V95% higher than 98.4, 95.5, and 99.75% led to statistically better OS, LC, and DFS, respectively; D99% higher than 63.8, 63.8, and 67.5 Gy guaranteed statistically better OS, LC, and DFS, respectively.

Another significant RT parameter for LC was GTVT (HR = 3.07 and *p* = 0.015), which was dichotomized to its third quartile. Patients with GTVT bigger than 43.2 cm^3^ had worse LC (Supplementary Figure 1). As mentioned before, all outcomes were significantly better with VMAT compared to step-and-shoot IMRT (Supplementary Figure 1).

Among clinical parameters, we found overall stage and T stage as significant prognosticators for LC and DFS, respectively (Supplementary Figure 1).

In addition, we found significant differences for patients treated with different RT modalities and for patients with different T stages. Table 3 shows the corresponding actuarial rates at 5 years for LC, DFS, and OS.

**Table 3:** Statistical values of KM analysis for the three outcomes: survival rates, hazard ratios, and *p*-values.

**Parameter**		**LC (%)**	**LC stat**.	**DFS (%)**	**DFS stat**.	**OS (%)**	**OS stat**.
T1–T2–T3	98/137	93.9	HR = 5.64, *p* < 0.001	81.6	HR = 2.36, *p* = 0.017	88.9	HR = 2.04, *p* = 0.12
T4	39/137	71.8		64.4		79.5	
VMAT	64/137	95.3	HR = 3.76, *p* = 0.027	89.1	HR = 3.02, *p* = 0.007	96.9	HR = 7.35, *p* = 0.03
IMRT	73/137	80.8		65.8		76.7	

**Figure 3:**
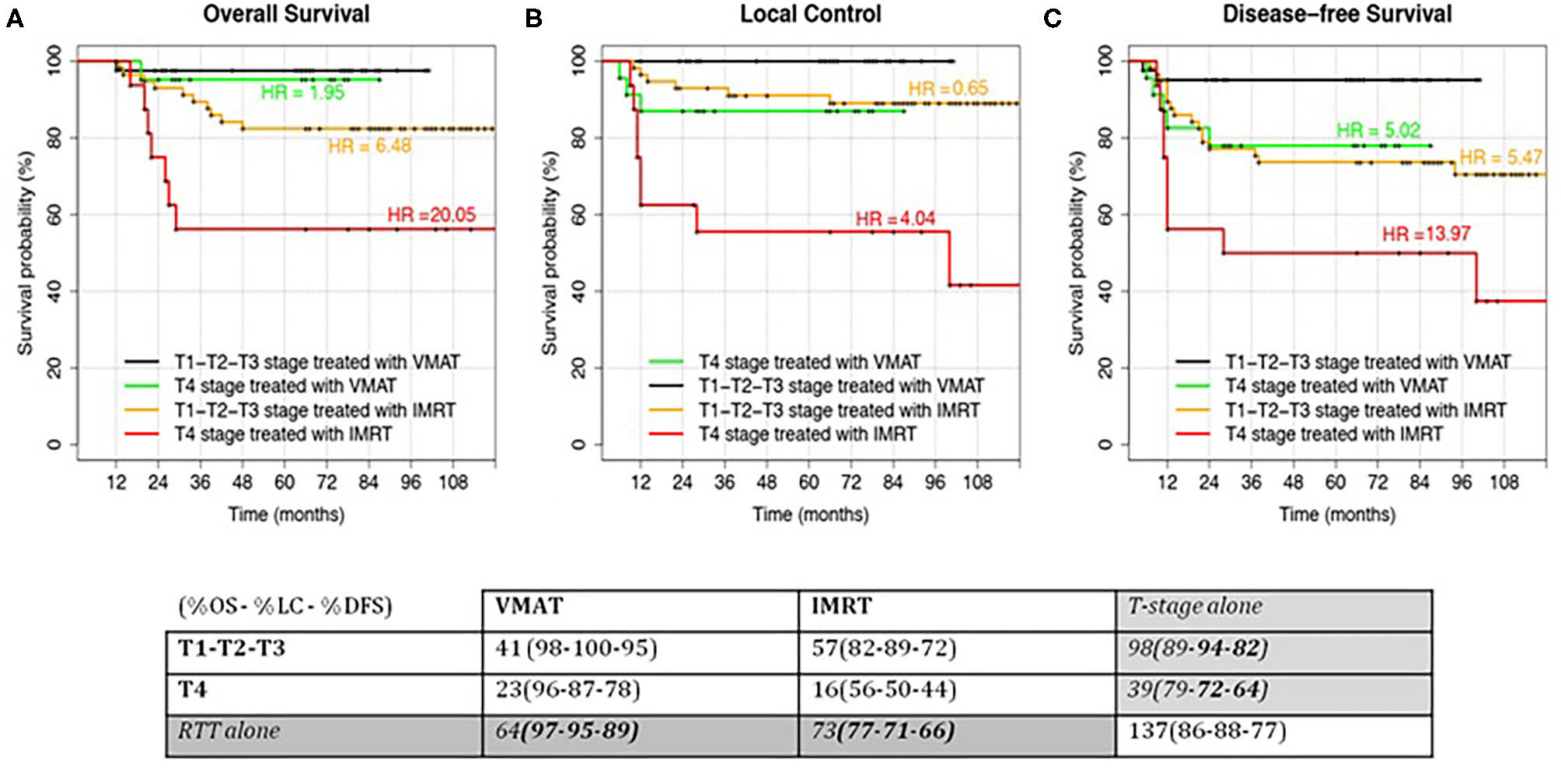
Kaplan–Meier curves for the analyzed outcomes: Overall Survival **(A)**, Local Control **(B)** and Disease-free Survival **(C)**. Patients were stratified according to the T-stage (T1–T2–T3 vs T4) and RT modality (IMRT vs VMAT). Hazard Ratio (HR) for the comparison of each group with the low-risk class (T1–T2–T3 treated with VMAT) were reported. In Local Control, HR were computed using T4 treated with VMAT as reference since no events were recorded in the low-risk class. HR = 1.95 and HR = 0.65 had a *p*-value > 0.05. HR = 5.02 had a *p*-value < 0.05. All the other HRs had a *p*-value > 0.01.

As a consequence, we decided to perform a subanalysis of the survival curves as a function of T stage (T1–T2–T3 vs. T4) and RT technique (VMAT vs. IMRT). A new combined variable, obtained from the two parameters, was studied in terms of survival: the results for the four groups are shown in Figure 3. Forty-one T1–T2–T3 and 23 T4 stage patients were treated with VMAT, while 57 T1–T2–T3 and 16 T4 stage patients were treated with IMRT. In particular, T4 stage patients treated with VMAT had similar survival rates compared to patients with T1–T2–T3 stages treated with step-and-shoot IMRT.

The group of T1–T2–T3 stage patients treated with VMAT (the group with best outcomes) was considered as reference for HR computing in the other patients' groups. This was not possible for LC (Figure 3B), where the reference group did not have any event (making it impossible to compute HR). However, it is easily understandable that HRs should be similar (but higher) to the OS ones. For this specific case, T4 stage patients treated with VMAT were considered as reference in HR computing.

Moreover, we can see how the curves of intermediate groups (T1–T2–T3 stages treated with IMRT and T4 stage treated with VMAT) are similar in LC and DFS but not in OS.

**Figure 4:**
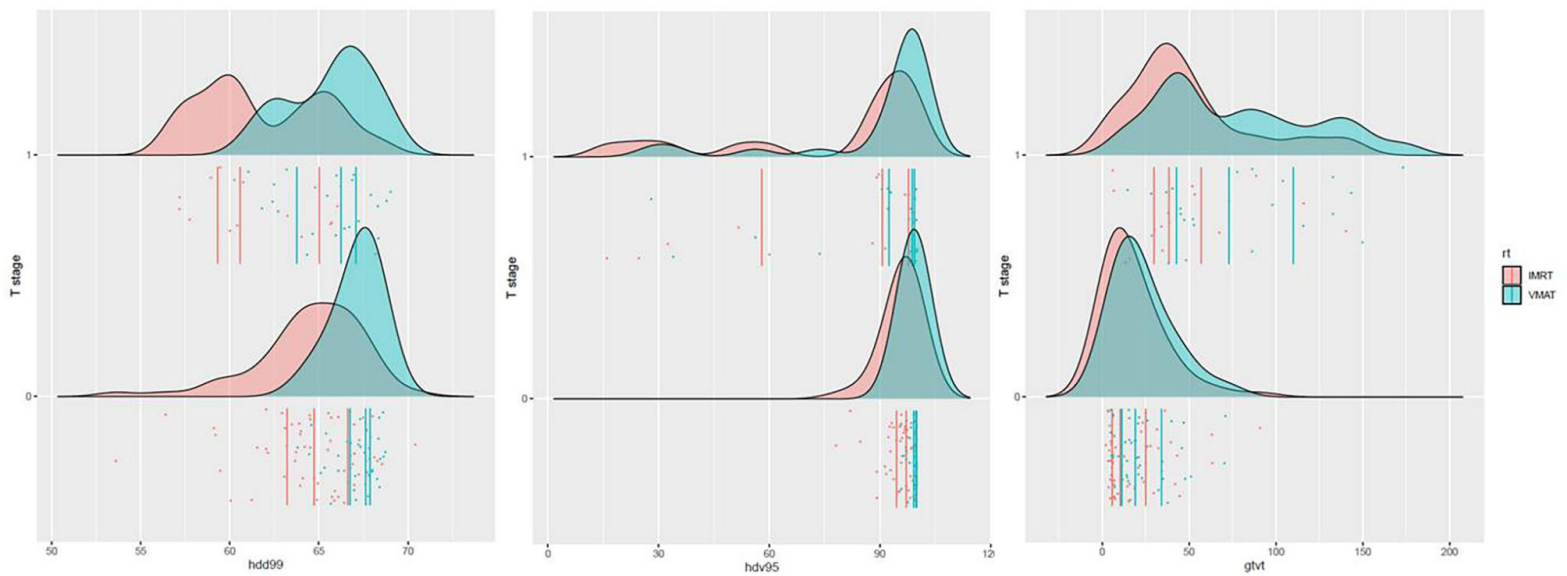
Distribution of D99, V95%, and GTVT. Patients were stratified according to the T stage (T1–T2–T3 = 0 vs. T4 = 1) and RT modality (IMRT vs. VMAT).

The distribution of the significant dose–volume parameters for the four groups is shown in Figure 4.

Finally, we decided to work with a multivariable model which took into account continuous (dosimetric or volumetric variables) or ordinal variables (stage and T stage). Due to the limited number of events, we tested all the possible models with two covariates that were found significant in univariate analysis (see Supplementary Materials). A bivariate model including GTVT and HDPTV D99% was found for LC. Hazard ratios for GTVT and HDPTV D99% as continuous variables in the bivariate model were 1.01 (*p*-value 0.04) and 0.88 (*p*-value 0.04), respectively. Area under the ROC curve for this model was 0.74, while it was 0.68 for the two univariate models with GTVT or HDPTV D99% (HRs were 1.01 with a *p*-value of 0.01 and 0.86 with a *p*-value of 0.01).

**Figure 5:**
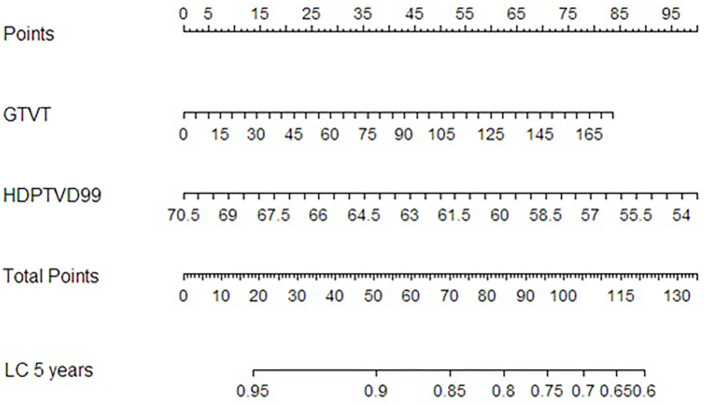
Nomogram for local control at 5 years after RT derived from the Cox proportional hazard regression model with GTVT and HDPTV D99% as variables.

A nomogram for the risk of LC at 5 and 8 years (Figure 5) was derived from the two-variable Cox regression model.

## Discussion

To our knowledge, this series had the longest follow-up for an NPC patient cohort in a low-incidence area.

Clinical outcomes were consistent with NPC series treated with IMRT and CHT reported in literature in endemic areas (6–8). Au and colleagues reported 5- and 8-year LC of 88.7% and 85.8%, progression-free survival (PFS) of 70.2 and 62.6%, OS of 78.2 and 68.5%, respectively. Sun et al. analyzing the prognostic factors in a series of 868 NPC patients showed 5-year disease-specific survival (DSS), local recurrence-free survival (LRFS), and PFS rates up to 84.7, 91.8, and 77.0%, respectively. As in these series, we confirmed the adverse prognostic role of advanced overall stage and T4 stage. However, treatment-related factors as a potential prognosticator on predicting outcome are less investigated.

Indeed, it is well-known that NPC is highly sensitive to ionizing radiation and total RT doses normally regarded as tumoricidal, allowing adequate LC and survival are ≥66 Gy (19); nonetheless, the correlation between precise dose–volume factors and clinical results has not been sufficiently investigated yet.

In the present analysis, we were able to demonstrate a prognostic impact of specific dose–volume parameters generated by IMRT techniques on long-term outcome for a homogeneous NPC patient cohort, although retrospectively. A better target coverage with higher values of V95 and D99% relative to HDPTV proved to significantly improve 5-year LC, DFS, and OS. Ng et al. in their study of 444 NPC patients, showed that underdosing a small portion, 3.4 cm^3^, of the primary GTV (GTVT), because of its proximity to critical structures, correlated with a worse outcome in terms of LC and DFS (19). Analogously, when analyzing the prognostic factors in a series of 868 NPC patients, Sun et al. concluded that the minimum dose to the GTV (which could be interpreted as D99%) of at least 65.6 Gy was a prognostic factor of LRFS, PFS, and DSS (8).

Interestingly and contrarily to the previous series, we found different D99% cutoffs for DFS compared to OS and LC. This could probably be explained by the impact of advanced lymph nodal disease on distant metastases and/or by the difference in delineation procedure, because they considered only primary tumor and retropharyngeal lymph nodes while we considered HDPTV encompassing also neck nodal disease.

Recently, a panel of experts in head and neck RT published a guideline on dose prioritization and acceptance criteria for NPC (20). They recommended a minimum dose to the GTV of at least 68.6 Gy, with an acceptable minimum dose set at 66.5 Gy, in line with Ng et al. and PTV coverage with ≥95% of the PD to the entire volume, or ≥93% of the PD to at least 99% of the volume.

IMRT is a major breakthrough in the treatment of NPC. It has been refined over time, till the evolution in VMAT, allowing the radiation dose to be efficiently delivered using a dynamic modulated arc. It is capable of producing highly conformal dose distributions with steep dose gradients and complex isodose surfaces, so as to improve target coverage and OAR sparing in cancers of the oropharynx, hypopharynx, and larynx (21). Indeed, in our previous works, we found a better target coverage with VMAT compared to conventional IMRT, in particular linked to a higher value of V100% (17, 22). This can explain why in our analysis the patients receiving VMAT had better 5-year LC, DFS, and OS compared to conventional IMRT. This result however has to be taken with caution, considering that in our institution, step-and-shoot IMRT has been employed up to 2009 and VMAT technique thereafter and that a learning curve exists in the use of IMRT for head and neck cancer. The evolution in imaging techniques to better define disease extension and a higher use of CHT should also be taken into account.

Another significant finding of our work was the prognostic value of GTVT on the LC trend and the identification of a volume point of GTVT for the prediction of LC at 5 years (43.2 cm^3^). This represents the first data in a low-incidence region. The GTVT meaning on the prognosis of NPC patients treated with IMRT has been extensively debated in several studies from endemic areas, with impact on different outcome endpoints probably due to the heterogeneity of samples and different imaging systems to define GTVT. Our finding is in line with data from endemic regions. In NPC, Feng et al. found that a large GTVT is a negative prognostic factor for LRC at 5 years (RR = 2.454, *p* = 0.002), with a 40-ml cutoff (23). Analyzing 321 patients with NPC, Wu et al. found a statistically significant correlation between GTVT and LC, DM, DFS, and OS (all *p* < 0.05) at univariate and multivariate analyses. According to the ROC curve analysis, the two cutoffs of 49 and 19 ml of GTV T were determined for LC and distant control, respectively (24). He et al. came to similar conclusions (25) when they found GTVT >46.4 ml to be an independent unfavorable prognostic indicator for OS, LRFS, DMFS, and DFS after IMRT in locally advanced NPC patients, with a prognostic value superior to the T category. We were not able to find a significant correlation between nodal GTVs and outcomes: no data have been published in literature on this topic, suggesting that major outcome prognosticators are overall stage and N stage (6, 8).

To come up with a useful approach to predict the risk of local recurrence at 5 years, so as to facilitate personalized management of NPC patients, we constructed a nomogram which combined the GTVT with D99% relative to HRPTV. This nomogram could help clinicians with decision making, enabling them to perform inexpensive, earlier identification of NPC patients at high risk of local recurrence after IMRT. Several previous studies have developed nomograms for individual local recurrence risk assessment in NPC patients based on clinical and radiomic variables. For example, Zhang et al. built a nomogram including age, body mass index (BMI), GTVT, and ethmoidal sinus invasion (26), whereas Chen et al. (27) included age, the neutrophil/leukocyte ratio, pathological type, GTVT, maxillary sinus invasion, ethmoidal sinus invasion, and lacerated foramen invasion. Another nomogram including gender, age, hemoglobin, N stage, and radiomic features has been proposed by Zhang et al. (28). A novelty of our nomogram is the integration of a specific dose–volume parameter (D99%) with a clinical variable (GTVT): this could turn out to be useful in critical T4 cases in which the proximity to surrounding structures could compromise target coverage. When evaluating an RT plan, in particular in those with a larger GTV abutting critical structures, a greater effort in plan optimization (taking care that D99% is at least equal to 63.8 Gy) could play an important role in terms of outcome. When that is not possible, maybe a different approach considering mixed beam therapy could be evaluated (e.g., proton boost). However, this research is retrospective, and our sample size limited. Thus, our nomogram still requires further validation; we will validate its efficiency in other NPC patient cohorts in the following studies.

For T4 patients, to overcome difficulties in dose optimization during planning when the target volume abuts critical structures and reduce late toxicities, a change in the standard practice of contouring the GTVT on pre-iCHT MRI is under investigation. Recent papers have reported the outcomes of NPC patients treated with IMRT, defining the gGTV on post-iCHT MRI, so as to reduce the volume. Yang et al. observed no significant differences in 1-, 2-, and 3-year OS, PFS, LRFFS, and DMFS with volume reduction after iCHT (29). Analogously, the experiences of Zhao et al. (30) and Xue et al. (31) reported good results in terms of disease control with mild toxicities. The dose–volume parameters to take into account in these situations are still to be defined and constitute an interesting field for future research.

We acknowledge the limitations of our study, primarily the retrospective nature of the analysis and the small sample size, although in a low-incidence region. Another limitation was that we did not discriminate the dosimetry only for lymph nodal disease and primary tumor alone. Finally, in our nomogram, we focused only on dose–volume parameters without considering clinical and biochemical data, as other authors did (29–31).

## Conclusions

In our analysis, we demonstrated the prognostic value of some dose–volume parameters, although in a retrospective series. The identification of a precise relationship between IMRT/VMAT plan results and clinical outcome is of paramount importance to finally establish dose–volume parameters serving as planning goal templates not only for routine clinical practice, within an institutional RT quality assurance program, but also for designing prospective NPC trials. In addition, for the first time, in a non-endemic area, a threshold value of GTVT, prognostic for LC, has been confirmed. Finally, to predict the risk of local recurrence at 5 years, we constructed a nomogram which combined the GTVT with D99% relative to HRPTV.

## Data Availability Statement

The datasets generated for this study are available on request to the corresponding author.

## Author Contributions

NI and EO conceived and designed the research. NI and ACa collected the data. NI, EO, ACi, and ACa analyzed and interpreted the data. NI, EO, ACi, and ACa prepared and wrote the manuscript. All authors commented on the manuscript and have given their final approval for submission. All authors contributed to the article and approved the submitted version.

## Conflict of Interest

The authors declare that the research was conducted in the absence of any commercial or financial relationships that could be construed as a potential conflict of interest.
