# Supplementary material for: Role of IMRT/VMAT-Based Dose and Volume Parameters in Predicting 5-Year Local Control and Survival in Nasopharyngeal Cancer Patients
Source: Front Oncol. 2020 Sep 24;10:518110. doi: 10.3389/fonc.2020.518110 (PMC7541899; doi:10.3389/fonc.2020.518110)
Supplement: Supplementary file 1 [file Data_Sheet_1.docx]

Supplementary Material

**Table S1**: t-test results for parameters selection. Variables with p-value <0.05 (highlighted in gray) were considered for Kaplan-Meier analysis.

| Variable | OS | DFS | LC |
| --- | --- | --- | --- |
| GTVT (cc) | 0.15 | 0.18 | 0.01 |
| GTVN (cc) | 0.24 | 0.19 | 0.45 |
| GTVNRP (cc) | 0.11 | 0.45 | 0.47 |
| GTVNNRP (cc) | 0.15 | 0.18 | 0.44 |
| HDPTV V95% (%) | < 0.01 | 0.01 | < 0.01 |
| HDPTV V100% (%) | 0.02 | 0.12 | 0.01 |
| HDPTV D1% (Gy) | 0.19 | 0.11 | 0.34 |
| HDPTV D99% (Gy) | < 0.01 | 0.02 | < 0.01 |
| HDPTV Dm (Gy) | 0.04 | 0.17 | 0.01 |
| HDPTV (cc) | 0.35 | 0.17 | 0.37 |

**Table S2:** distribution of the dosimetric variables selected for Kaplan-Meier analysis in terms of mean value and standard deviation, stratified according to OS, DFS and LC.

| **Outcome** | **Variable** | **Outcome =0**  **at 5 years**  (mean±SD) | **Outcome =1**  **at 5 years**  (mean±SD) | **t-test**  **p-value** |
| --- | --- | --- | --- | --- |
| **OS** | HDPTV V95% (%) | 95.3 ± 12.1 | 82.5 ± 24.3 | <0.01 |
|  | HDPTV V100% (%) | 62.0 ± 23.4 | 49.7 ± 30.2 | 0.02 |
|  | HDPTV D99% (Gy) | 65.5 ± 2.9 | 63.1 ± 2.9 | <0.01 |
|  | HDPTV Dm (Gy) | 70.4 ± 1.6 | 69.7 ± 2.4 | 0.04 |
| **DFS** | HDPTV V95% (%) | 95.0 ± 12.7 | 88.2 ± 20.7 | 0.01 |
|  | HDPTV D99% (Gy) | 65.4 ± 2.9 | 64.2 ± 3.2 | 0.02 |
| **LC** | GTVT (cc) | 31.0 ± 33.0 * | 52.5 ± 43.0 * | 0.01 |
|  | HDPTV V95% (%) | 95.0 ± 12.5 | 81.6 ± 25.4 | <0.01 |
|  | HDPTV V100% (%) | 61.9 ± 23.3 | 47.0 ± 31.8 | 0.01 |
|  | HDPTV D99% (Gy) | 65.4 ± 2.9 | 63.1 ± 3.5 | <0.01 |
|  | HDPTV Dm (Gy) | 70.4 ± 1.6 | 69.3 ± 2.6 | 0.01 |
| GTVT distribution was considered as Gaussian even if it is not. The variable ranged from 2.2 to 173.3 cc and, despite the resulting standard deviation, it never assumed negative values. | | | | |


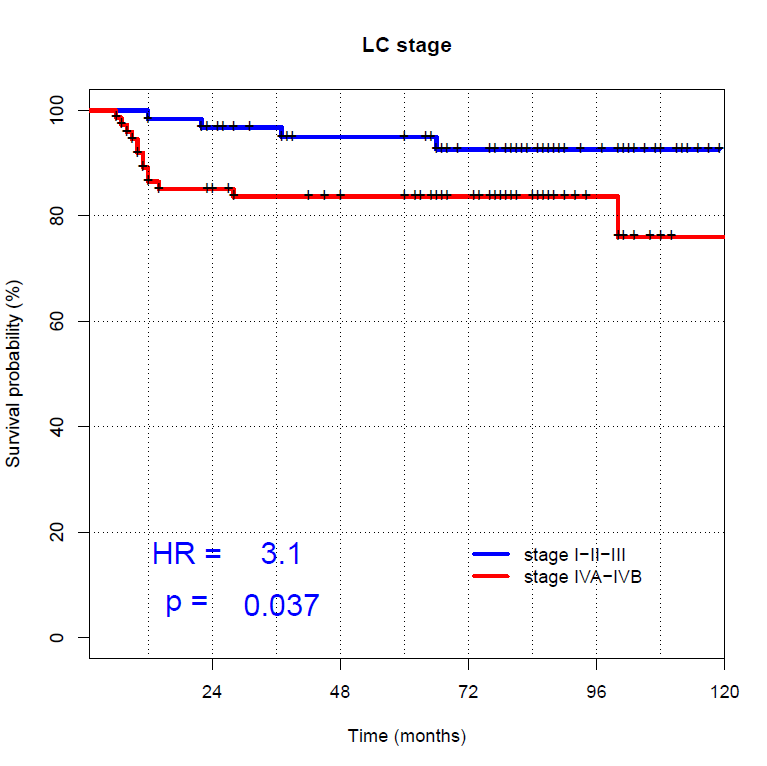

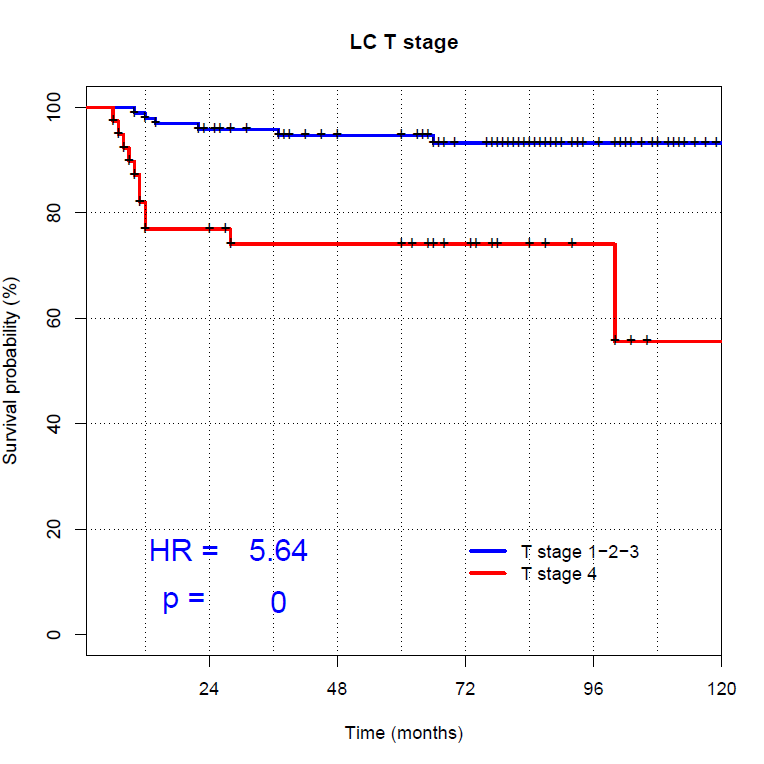


**Figure S1:** Kaplan-Meier curves of every significant factor in each clinical outcome

**Table S3**: univariate analysis results for LC at 5 years. Variables with p-value <0.05 are highlighted in gray.

| **Variable** | **Coeff.** | **Std. Err.** | **z-score** | **P>\|z\|** | **OR** |
| --- | --- | --- | --- | --- | --- |
| **GTVT** | 0.013566 | 0.006313 | 2.148809 | 0.03165 | 1.013659 |
| **GTVN** | -0.00078 | 0.005965 | -0.13029 | 0.896339 | 0.999223 |
| **GTVNRP** | -0.00296 | 0.034799 | -0.08517 | 0.932129 | 0.997041 |
| **GTVNNRP** | -0.00091 | 0.00631 | -0.14387 | 0.8856 | 0.999093 |
| **HDPTV V95%** | -0.03514 | 0.01266 | -2.77596 | 0.005504 | 0.965468 |
| **HDPTV V100%** | -0.02178 | 0.010157 | -2.14435 | 0.032005 | 0.978455 |
| **HDPTV D1%** | -0.04872 | 0.113938 | -0.42758 | 0.668956 | 0.95245 |
| **HDPTV D99%** | -0.21016 | 0.079112 | -2.65642 | 0.007897 | 0.810459 |
| **HDPTV DMean** | -0.34732 | 0.145446 | -2.38795 | 0.016943 | 0.706581 |
| **HDPTV** | -0.00069 | 0.00201 | -0.34359 | 0.731154 | 0.99931 |
| **T_dico=1** | 1.858451 | 0.587567 | 3.162958 | 0.001562 | 6.413793 |
| **Stage_dico=1** | 1.353505 | 0.67042 | 2.018892 | 0.043498 | 3.870968 |
| **N_dico=1** | 0.024693 | 0.580685 | 0.042523 | 0.966082 | 1.025 |
| **RT_dico=1** | 1.386294 | 0.670413 | 2.067822 | 0.038657 | 4 |

**Table S4**: univariate analysis results for DFS at 5 years. Variables with p-value <0.05 are highlighted in gray.

| **Variable** | **Coeff.** | **Std. Err.** | **z-score** | **P>\|z\|** | **OR** |
| --- | --- | --- | --- | --- | --- |
| **GTVT** | 0.005018 | 0.005549 | 0.904393 | 0.365787 | 1.005031 |
| **GTVN** | 0.003432 | 0.003957 | 0.867304 | 0.385776 | 1.003438 |
| **GTVNRP** | 0.002894 | 0.025225 | 0.114729 | 0.90866 | 1.002898 |
| **GTVNNRP** | 0.003795 | 0.004112 | 0.922921 | 0.356048 | 1.003802 |
| **HDPTV V95%** | -0.02444 | 0.011971 | -2.04173 | 0.041178 | 0.975855 |
| **HDPTV V100%** | -0.00935 | 0.0081 | -1.15372 | 0.248613 | 0.990698 |
| **HDPTV D1%** | 0.09949 | 0.082863 | 1.200655 | 0.229885 | 1.104607 |
| **HDPTV D99%** | -0.12348 | 0.064267 | -1.92133 | 0.04069 | 0.883841 |
| **HDPTV DMean** | -0.11228 | 0.1169 | -0.96047 | 0.336816 | 0.893794 |
| **HDPTV** | 0.001388 | 0.001446 | 0.959837 | 0.337137 | 1.001389 |
| **T_dico=1** | 0.868089 | 0.431919 | 2.009842 | 0.044448 | 2.382353 |
| **Stage_dico=1** | 1.06769 | 0.455924 | 2.341814 | 0.01919 | 2.908654 |
| **N_dico=1** | 0.763215 | 0.423491 | 1.802199 | 0.071514 | 2.145161 |
| **RT_dico=1** | 1.320612 | 0.473159 | 2.791055 | 0.005254 | 3.745714 |

**Table S5**: univariate analysis results for OS at 5 years. Variables with p-value <0.05 are highlighted in gray.

| **Variable** | **Coeff.** | **Std. Err.** | **z-score** | **P>\|z\|** | **OR** |
| --- | --- | --- | --- | --- | --- |
| **GTVT** | 0.006453 | 0.006293 | 1.025362 | 0.305192 | 1.006474 |
| **GTVN** | 0.003213 | 0.004542 | 0.707371 | 0.479336 | 1.003218 |
| **GTVNRP** | -0.05785 | 0.049037 | -1.17974 | 0.238103 | 0.943791 |
| **GTVNNRP** | 0.004614 | 0.004546 | 1.014934 | 0.310137 | 1.004624 |
| **HDPTV V95%** | -0.0365 | 0.012636 | -2.88854 | 0.00387 | 0.964157 |
| **HDPTV V100%** | -0.01841 | 0.009334 | -1.97264 | 0.048537 | 0.981755 |
| **HDPTV D1%** | 0.08449 | 0.097182 | 0.869401 | 0.384628 | 1.088162 |
| **HDPTV D99%** | -0.22017 | 0.074361 | -2.96089 | 0.003068 | 0.80238 |
| **HDPTV Dm** | -0.23112 | 0.135203 | -1.70946 | 0.087365 | 0.793641 |
| **HDPTV** | 0.000695 | 0.001734 | 0.400686 | 0.688651 | 1.000695 |
| **T_dico=1** | 0.713467 | 0.50957 | 1.400136 | 0.161473 | 2.041056 |
| **Stage_dico=1** | 0.995718 | 0.552573 | 1.801967 | 0.071551 | 2.706667 |
| **N_dico=1** | 0.205444 | 0.514788 | 0.399085 | 0.689831 | 1.22807 |
| **RT_dico=1** | 2.241849 | 0.769941 | 2.911714 | 0.003595 | 9.410714 |
